# Supplementary material for: Research on factors affecting the spread of dust pollution in conveyor belt workshop and research on wet dust reduction technology
Source: PLoS One. 2024 Feb 23;19(2):e0299328. doi: 10.1371/journal.pone.0299328 (PMC10889843; doi:10.1371/journal.pone.0299328)
Supplement: Attachment — Submitted filename: Responses to Technical Check Results.docx [file pone.0299328.s001.docx]

Dear Editors and Reviewers:

Thank you for your letter and for the reviewers’ comments concerning our manuscript entitled “Research on factors affecting the spread of dust pollution in conveyor belt workshop and research on wet dust reduction technology” (ID: PONE-D-23-38475). All comments are very important and they are great support for our scientific research work. We have studied comments carefully and have made correction **(Revised portion are marked in red in the text)**. The main corrections in the paper and the responds to the reviewer’s comments are as flowing:

**Reviewer 1**

The article investigates the airflow and dust transport characteristics of parallel conveyor belt systems in coal preparation plants under different induced airflow conditions, focusing on dust The spray dedusting system is designed and implemented according to the pollution law of air flow, and the movement law of droplets after the spray system is arranged in the workshop is simulated and analyzed. The field test results show that the implementation of the dedusting system has achieved good results. The research content, methods, conclusions, and on-site applications of the article have good innovation and practical significance, and can provide a theoretical basis for dust pollution control in belt conveyor systems similar to coal preparation plants.

Answers:Thank you for your professional evaluation of my manuscript, for your work I am deeply grateful, I will seriously listen to your suggestions to improve the article, I wish you a happy life!

**Reviewer 2**

The subject of the study is the dust contamination mechanism under changing conditions of wind flow field in the workshop of a coal processing plant, which was investigated using a computational fluid dynamics based approach. The effects of induced airflow and corridor wind speed on the internal airflow field of the workshop were investigated, and the dust pollution mechanism under the changing conditions of the workshop wind field of the coal power plant was derived. The results of this study can provide an important reference for the study of dust pollution control in the workshop of coal processing plant. However, there are some minor problems in the article, which need to be slightly modified, and I support the publication of this paper.

Answers: Thank you very much for the excellent and professional revisions of our manuscript. We have studied the reviewer's comments carefully and have made revisions that have been marked in red in the revised manuscript.

**Comment 1**)In this paper, all variables in Equations 4, 5, 6, and 7 should be interpreted.

Answers: Thank you for your comments.I have changed the variables to the correct format .

**Comment 2**)In Fig. 3, the curve represents the wind flow velocity, and the location of the measurement point of the curve should be labeled in the model plot.

Answers: Thank you for your comments.I have modified Figure 3 to mark the location of the measurement points

**Comment 3**)On page 10 of the article, in Table 1, the boundary conditions are incomplete, please add them.

Answers: Thank you for your comments.I have added to the conditions

**Comment 4**)Throughout the article, the authors simulated the selection of 3 sets of corridor wind speeds as well as 3 sets of induced airflow values, please explain the reasons for the selections made.

Answers: Thank you for your comments.The authors chose the three sets of wind speeds mainly based on the maximum and minimum wind speeds obtained from the actual measurements in the field to be used as the simulation conditions, and the average values of the measured data as the study conditions.

**Comment 5**) Fig 5, 6, 7, and 8 are not formatted correctly, and the figure numbers should not be within the pictures.

Answers: Thank you for your comments.Figure has been revised as requested and labeled in the article

**Comment 6**) This paper studies the coal preparation plant workshop spray dust reduction, but the authors for the selection of nozzles based on the selection and selection of models is not given, should be supplemented in the text.

Answers: Thank you for your comments.The dust reduction system uses pneumatic atomized nozzles, which are more suitable for capturing fine dust.

**Comment 7**) Fig 9 is not clear enough, please ask the author to adjust and modify it.

Answers:Thank you for your comments.Fig 9 has been adjusted in accordance with your suggestions.

**Comment 8**) Conclusion 2 of the article is not expressed with enough precision and summarized with enough fleshing out.

Answers: Thank you for your comments. I have added and summarized the conclusions based on your suggestions, which are much appreciated.

Thank you very much for your suggestion!

**Reviewer 3**

The article describe CFD simulations of dust distribution in a conveyor belt workshop. Mitigation measures using water sprays are also considered.The introduction gives a good overview of the problem with dust, and of previous simulation studies of the problem. The computational methods should be better described, see comments below. The result section has a good description of the results, but the images need improvement. Overall, the study demonstrates a nice use of computational methods to improve working environment. I recommend publication of the study, considering the following comments are taken into account by the authors:

Answers:Thank you for your professional evaluation of my manuscript, I have seriously revised your suggestions on my manuscript, your suggestions make my manuscript better, thank you for your guidance on my work, I wish you a happy life!

**Comment 1**) The abstract should contain a short sentence with background on the problem.

Answers: Thank you for your comments.I have revised the abstract as you suggested.

**Comment 2**) Equations for the turbulence model come a bit suddenly and out of context. Please first write the RANS Navier-Stokes equations and explain the connection to the turbulence model.

Answers: Thank you for your comments. The mathematical modeling equations section has been supplemented and refined.The RANS Navier-Stokes equations have been added.

**Comment 3**) Explain the boundary conditions better, please include a figure showing inlets, outlets, walls etc.

Answers: Thank you for your comments.The conditions of the model's inlets, outlets, and walls are described in additional detail in Manuscript 3.1.

**Comment 4**) What do you mean by cell mass values in the grid setup, and how does this relate to grid quality?

Answers: Thank you for your comments. Grid cell quality between 0.4 and 1 indicates that the set grid quality is good and meets the calculation requirements

**Comment 5**) What do you mean by “the airflow velocity in grid B already remains constant”, the velocity is clearly not constant?

Answers: Thank you for your comments.Grid B's airflow velocity has reached stabilization is very little affected by the grid, the previous inaccurate description has been revised in the article, thank you for your guidance.

**Comment 6**) Fig 3, please have a better caption, the caption should explain the figure without needing to go into the text. The grid pictures should be bigger.

Answers: Thank you for your comments. Fig 3 has been modified

**Comment 7**) In the text, you do not explain where this velocity in Fig 3 is extracted from?

Answers: Thank you for your comments.The speed measurement points have been labeled in the Fig 3.

**Comment 8**) For the validation study, please give the exact coordinates of the measurement points. Also explain what equipment was used for the measurements.

Answers: Thank you for your comments. Additional description of manuscript paragraph 3.4, location of measurement points and method of measurement.

**Comment 9**) Fig 5-9, these should be bigger, they are very hard to interpret. Also, the captions should be more descriptive.

Answers: Thank you for your comments. Changes have been made to Fig 5-9.

**Comment 10**) The positions of the water mist devices should be clearly indicated.

Answers: Thank you for your comments.A diagram of the location of the water mist unit has been added to the manuscript.（Fig 10）

Thank you very much for your suggestion!
