# Supplementary material for: Research on factors affecting the spread of dust pollution in conveyor belt workshop and research on wet dust reduction technology
Source: PLoS One. 2024 Feb 23;19(2):e0299328. doi: 10.1371/journal.pone.0299328 (PMC10889843; doi:10.1371/journal.pone.0299328)
Supplement: Attachment — Submitted filename: Responses to Technical Check Results 1.docx [file pone.0299328.s002.docx]

Dear Editors and Reviewers:

Thank you for your letter and for the reviewers’ comments concerning our manuscript entitled “Research on factors affecting the spread of dust pollution in conveyor belt workshop and research on wet dust reduction technology” (ID: PONE-D-23-38475). All comments are very important and they are great support for our scientific research work. We have studied comments carefully and have made correction **(Revised portion are marked in red in the text)**. The main corrections in the paper and the responds to the reviewer’s comments are as flowing:

**Reviewer 2**

The subject of the study is the dust contamination mechanism under changing conditions of wind flow field in the workshop of a coal processing plant, which was investigated using a computational fluid dynamics based approach. The effects of induced airflow and corridor wind speed on the internal airflow field of the workshop were investigated, and the dust pollution mechanism under the changing conditions of the workshop wind field of the coal power plant was derived. The results of this study can provide an important reference for the study of dust pollution control in the workshop of coal processing plant. However, there are some minor problems in the article, which need to be slightly modified, and I support the publication of this paper.

Answers: Thank you very much for the excellent and professional revisions of our manuscript. We have studied the reviewer's comments carefully and have made revisions that have been marked in red in the revised manuscript.

**Comment 1**)However, equations 2, 3 and 4 still need further revision. Equation 2 is not the continuity equation, it is the momentum equation, and it's not written correctly. Equations 3 and 4 are not consistent, equation 3 has the transient term, while equation 4 does not. Also, the convection term is written differently. Please also separate vector variables from scalars, either with an arrow or bold font.

Answers: Thank you for your comments.I have changed the variables to the correct format .Your advice to my academic play a guiding role, for the formula of the understanding I still have deficiencies, but after my study and ask for advice has been in accordance with your requirements for modification, thank you for your teaching!

Thank you very much for your suggestion!
